# Diagnostic accuracy of self-reported age-related macular degeneration in the ASPREE Longitudinal Study of Older Persons

**DOI:** 10.1038/s41433-023-02754-y

**Published:** 2023-09-20

**Authors:** Myra B. McGuinness, Liubov Robman, Lauren A. B. Hodgson, Cammie Tran, Robyn L. Woods, Alice J. Owen, John J. McNeil, Galina Makeyeva, Walter P. Abhayaratna, Robyn H. Guymer

**Affiliations:** 1 Centre for Eye Research Australia, Royal Victorian Eye and Ear Hospital, Melbourne, VIC Australia; 2 Centre for Epidemiology and Biostatistics, Melbourne School of Population and Global Health, University of Melbourne, Melbourne, VIC Australia; 3 Ophthalmology, Department of Surgery, University of Melbourne, Melbourne, VIC Australia; 4 Department of Epidemiology and Preventive Medicine, School of Public Health and Preventative Medicine, Monash University, Melbourne, VIC 3004 Australia; 5 College of Health and Medicine, The Australian National University, Canberra, ACT 0200 Australia

**Keywords:** Epidemiology, Eye manifestations

## Abstract

**Background:**

The validity of findings from epidemiological studies using self-report of ophthalmic conditions depends on several factors. We assessed the diagnostic accuracy of self-reported age-related macular degeneration (AMD) among older Australians enroled in a primary prevention clinical trial and compared diagnostic accuracy between demographic subgroups.

**Methods:**

At baseline (2010–2015), Australian sub-study participants of the ASPirin in Reducing Events in the Elderly (ASPREE) trial, underwent bilateral two-field, 45° non-mydriatic colour retinal photography. Beckman classification of any-stage AMD was used as the reference standard diagnosis. Participants were asked whether a doctor had ever diagnosed them with “macular degeneration” (the index test) via a paper-based questionnaire as part of the ASPREE Longitudinal Study of Older Persons (ALSOP) within the first year of enrolment.

**Results:**

In total, 4193 participants were included (aged 70–92 years, 50.8% female). Of those, 262 (6.3%) reported having AMD and 92 (2.2%) were unsure. Retinal grading detected 2592 (61.8%) with no AMD, 867 (20.7%) with early, 686 (16.4%) with intermediate and 48 (1.1%) with late AMD (*n* = 1601 with any-stage AMD, 38.2%). Self-reported AMD had 11.4% sensitivity (95% CI 9.9–13.1) and 96.9% specificity (95% CI 96.2–97.6) for any-stage AMD, with 69.8% and 63.9% positive and negative predictive values. Sensitivity was higher among participants with late-stage AMD (87.5%), older participants (26.8%), and those with poorer vision (41.0%).

**Conclusions:**

Although most participants with late-stage AMD were aware of having AMD, the majority with early and intermediate AMD were not. Therefore, findings from studies that rely on disease self-report should be interpreted with caution.

## Introduction

Large community-based studies can play an important role in capturing the level of ocular health in society and in identifying sectors of the population with additional eyecare needs. However, ocular diagnostic testing is not always feasible in studies primarily designed to investigate non-ocular conditions and participant-report of eye disease may be the most practical way to obtain that information [1–4]. Given findings from these studies can have implications for policy development, allocation of resources, and generation of hypotheses for future interventional research, it is important that the reliability of self-report be quantified for each condition and population of interest [5, 6].

Accurate self-report of ocular conditions is dependent on participants having the motivation and means to undergo a comprehensive eye exam, appropriate communication of findings by the eyecare provider, and correct recall of those details. Therefore, the diagnostic accuracy of self-report is likely to vary and may be influenced by factors such as access to eyecare, visual function, level of health literacy, and the presence of comorbid conditions [7].

Self-report has previously been shown to be unreliable for AMD with errors increasing with age, time since last eye exam, and poorer vision [7–9]. However, the extent to which diagnostic accuracy of self-reported AMD differs according to population characteristics has not been investigated in detail. Therefore, we aimed to investigate the diagnostic accuracy of questionnaire-based self-report of AMD among generally healthy older Australians using expert-graded bilateral two-field 45° colour fundus photography as the reference standard in this cross-sectional study with prospective and standardised data collection. We compared diagnostic accuracy between demographic and clinical subgroups to identify differences in the ability to self-report.

## Materials and methods

### Study design, eligibility, and recruitment

Australians aged ≥70 years were recruited into the ASPirin in Reducing Events in the Elderly (ASPREE) randomised placebo-controlled trial of 100 mg daily aspirin through general practices across five states/territories between 2010 and 2014 (clinicaltrials.gov registration number: NCT01038583) [10, 11]. Participants in the USA arm of the ASPREE trial were not invited to participate in the ASPREE-AMD or ASPREE Longitudinal Study of Older Persons (ALSOP) studies and therefore have not been included in this analysis. Exclusion criteria precluded enrolment of people with known cardiovascular disease, independence-limiting physical disability, anaemia, high risk of bleeding, dementia, uncontrolled high blood pressure, or ongoing use of antiplatelet/anticoagulant medication [10]. People who needed assistance to complete basic activities of daily living (eating, dressing, walking across a room, bathing, toileting and transferring) were excluded. Participants were required to be able to read and sign a consent form as part of the eligibility criteria and the use of visual aids was permitted for these tasks. Potential participants were also informed that they would be required to see well enough to complete selected written questionnaires confidentially without assistance from other individuals.

Retinal photography was conducted between March 2010 and January 2015 ranging from six months before to three months after the randomisation date as part of three ASPREE sub-studies: the ASPREE-AMD sub-study (Australian New Zealand Clinical Trial Registry: ACTRN12613000755730) [12], the Study of Neurocognitive Outcomes, Radiological and Retinal Effects of Aspirin in Sleep Apnoea (SNORE-ASA) sub-study (ACTRN12612000891820) [13], and the Aspirin for the prevention of cognitive decline in the Elderly: a Neuro-Vascular Imaging Study (ENVIS-ion, ACTRN12609000613202) [14]. Some ASPREE participants were enroled prior to the sub-studies commencing in their region and therefore were not invited to participate. The sample size of the ASPREE-AMD study was chosen to detect a difference in the rate of AMD progression between randomisation groups [12].

A paper-based medical questionnaire was mailed to Australian participants who were still active in the ASPREE study within the first year of enrolment (the majority between 3–6 months post randomisation) as part of ALSOP [3].

ASPREE and sub-studies were approved by the Monash University (2006/745MC, CF11/1100, CF11/1935, CF13/282, CF12/0367, CF08/1314), RACGP (NREEC 02/22b and 11258), University of Tasmania (H0008933), Australian National University (2008/100, 2008/115), ACT Health (ETH.11.07.997, ETH.11/07.998), University of Adelaide (H-250-2011) and Alfred Hospital (452/11, 79/08) Human Research Ethics Committees. Participants provided separate written informed consent for each sub-study. These studies were undertaken in accordance with the tenants of the Declaration of Helsinki and the National Health and Medical Research Council Statement on Ethical Conduct in Human Research [15].

### Demographic data and medical history

Age at randomisation, gender, race, primary language, country of birth, living situation, years of education, and area of residence were collected by study staff at the baseline visits of the ASPREE study. Area of residence was used to derive remoteness area (major city vs not major city) and the Index of Relative Socio-economic Advantage and Disadvantage (IRSAD) decile [16].

As part of the ALSOP baseline medical questionnaire, participants were asked if a doctor had ever diagnosed “Macular degeneration” and the response options were Yes, No, and Don’t know. They were also asked, “At the present time, how would you rate your eyesight? (with glasses or contact lenses, if you wear them).” Response options were Excellent, Good, Fair, Poor, Very poor, and Completely blind.

### Retinal photography and grading

Bilateral digital 45° macular- and disc-centred colour fundus photographs were captured using non-mydriatic fundus cameras (Canon Inc., Tokyo) with Digital Health Care software (UK) [12]. Digital images were viewed immediately and repeated if necessary. All images were graded by two senior graders from the Centre for Eye Research Australia while masked to participant characteristics and questionnaire responses. Images were viewed on high resolution monitors using FastStone Image Viewer software v6.5 (FastStone Corporation).

Per-person AMD status was assigned as the stage of disease in the worse affected eye according to the Beckman classification system [17]. Participants were classified as having no apparent ageing in the absence of drusen and retinal pigment epithelium abnormalities. Those with drusen <63 μm and no retinal pigmentary abnormalities were classed as having normal ageing changes. Early AMD was classified as medium drusen (≥63–<125 μm) with no pigmentary abnormalities, while intermediate AMD was defined as medium drusen with retinal pigmentary abnormalities, or large drusen (≥125 μm) with or without AMD pigmentary abnormalities. Late AMD was defined as either neovascular AMD (nAMD), or geographic atrophy (GA). All cases of late AMD were adjudicated by a retinal-specialist ophthalmologist [18].

### Statistical methods

Complete-case analyses were conducted, i.e., only participants with non-missing data on self-reported AMD and gradable retinal images in at least one eye were included in the analyses. Demographic variables were compared according to self-reported AMD status via Pearson’s chi-squared test.

Photograph-graded AMD status was used as the reference-standard diagnosis. For the primary analysis, AMD was diagnosed as the detection of any-stage AMD, i.e., early, intermediate, or late AMD (versus no AMD or normal ageing changes only). To compare with previous studies that have used self-report of vision-affecting AMD [8], the use of late AMD as a reference-standard (compared to those with no AMD/normal ageing, early AMD, or intermediate AMD) was also examined, as was intermediate AMD or worse (compared to those with no AMD/normal ageing or early AMD).

Self-report of AMD, the index test, was dichotomised as “aware” versus “not aware” of having AMD. Participants who responded ”Don’t know” (an inconclusive index test) were treated as not being aware of the condition (i.e., best-case scenario) [19]. Sensitivity analyses were conducted under the worst-case scenario (inconclusive index tests treated as positive responses) and after exclusion of participants with an inconclusive index test.

Sensitivity, specificity, positive and negative predictive values, positive and negative likelihood ratios, diagnostic odds ratio, and area under the receiver operating characteristic curve were estimated (see Supplementary Table 1 for formulae and interpretation). Interaction terms were included in mixed-effects logistic regression models to compare sensitivity and specificity between demographic subgroups.

Analyses were conducted using Stata/MP v17.0 (StataCorp LLC, College Station, TX).

## Results

### Participant characteristics

**Fig. 1:**
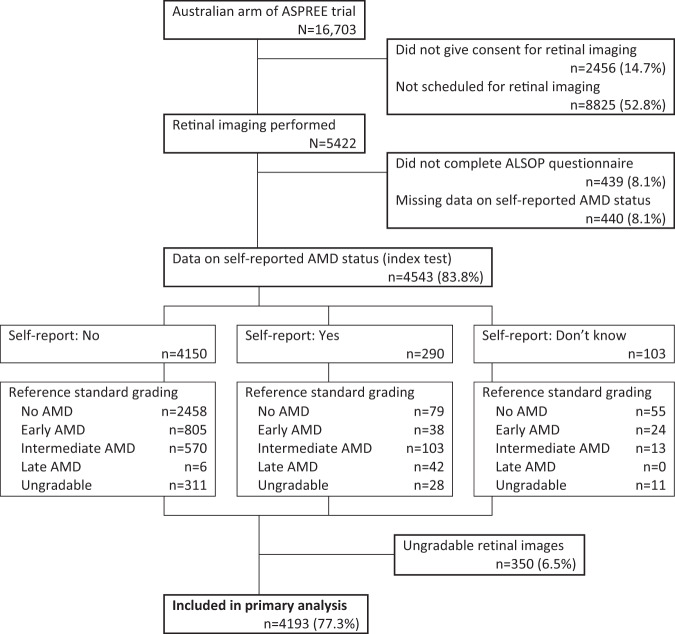
Study participation flow chart. AMD age-related macular degeneration, ASPREE ASPirin in Reducing Events in the Elderly trial, ALSOP ASPREE Longitudinal Study of Older Persons. Participants with inconclusive index text (“Don’t know”) were classified as reporting no AMD under the best-case scenario.

Among 16,703 Australian ASPREE participants, 5422 (32.5%) underwent retinal imaging. Of those, 879 (16.2%) did not have any data on self-reported AMD status and 350 (6.5%) did not have a gradable retinal photograph for at least one eye, leaving 4193 participants (77.3%, see Fig. 1). Included participants were aged 70–92 years (median 73 years, IQR 71–76) and 2129 (50.8%) were female (see Table 1). Australian ASPREE participants who were excluded from the current analysis were more likely to be older, female, have fewer years of education and live in an area with higher levels of disadvantage (see Supplementary Table 2).

**Table 1:** Participant characteristics according to self-reported age-related macular degeneration (AMD) status.

	Self-reported AMD, *n* (%)			Total	*p*-value^a^
	No	Yes	Don’t know		
	*n* = 3839	*n* = 262	*n* = 92	*N* = 4193	
Baseline age					<0.001
70–74	2557 (66.6%)	123 (46.9%)	58 (63.0%)	2738 (65.3%)	
75–79	900 (23.4%)	79 (30.2%)	21 (22.8%)	1000 (23.8%)	
80–84	299 (7.8%)	42 (16.0%)	10 (10.9%)	351 (8.4%)	
85+	83 (2.2%)	18 (6.9%)	3 (3.3%)	104 (2.5%)	
Gender					0.005
Male	1889 (49.2%)	116 (44.3%)	59 (64.1%)	2064 (49.2%)	
Female	1950 (50.8%)	146 (55.7%)	33 (35.9%)	2129 (50.8%)	
Race					0.373
White/Caucasian	3793 (98.8%)	262 (100.0%)	90 (97.8%)	4145 (98.9%)	
Asian	29 (0.8%)	0 (0.0%)	1 (1.1%)	30 (0.7%)	
>1 race/Other/Prefer not to say	17 (0.4%)	0 (0.0%)	1 (1.1%)	18 (0.4%)	
Primary language					<0.001
English	3701 (96.4%)	253 (96.6%)	81 (88.0%)	4035 (96.2%)	
Other than English	138 (3.6%)	9 (3.4%)	11 (12.0%)	158 (3.8%)	
Country of birth					0.040
Australia	2903 (75.6%)	199 (76.0%)	59 (64.1%)	3161 (75.4%)	
Outside Australia	936 (24.4%)	63 (24.0%)	33 (35.9%)	1032 (24.6%)	
Years of education					0.010
<9	484 (12.6%)	39 (14.9%)	23 (25.0%)	546 (13.0%)	
9–12	1560 (40.6%)	104 (39.7%)	34 (37.0%)	1698 (40.5%)	
>12	1795 (46.8%)	119 (45.4%)	35 (38.0%)	1949 (46.5%)	
Living situation					0.011
At home alone	1059 (27.6%)	95 (36.3%)	28 (30.4%)	1182 (28.2%)	
With family/friends/spouse	2772 (72.2%)	165 (63.0%)	64 (69.6%)	3001 (71.6%)	
In a residential/retirement home	8 (0.2%)	2 (0.8%)	0 (0.0%)	10 (0.2%)	
IRSAD decile					0.362
1–5 (lower levels of advantage)	1252 (32.6%)	77 (29.4%)	34 (37.0%)	1363 (32.5%)	
6–10 (higher levels of advantage)	2580 (67.2%)	185 (70.6%)	58 (63.0%)	2823 (67.3%)	
Missing	7 (0.2%)	0 (0.0%)	0 (0.0%)	7 (0.2%)	
Lives in a major city					0.814
No	1158 (30.2%)	78 (29.8%)	25 (27.2%)	1261 (30.1%)	
Yes	2674 (69.7%)	184 (70.2%)	67 (72.8%)	2925 (69.8%)	
Missing	7 (0.2%)	0 (0.0%)	0 (0.0%)	7 (0.2%)	
Self-rated eyesight					<0.001
Excellent	708 (18.4%)	17 (6.5%)	6 (6.5%)	731 (17.4%)	
Good	2517 (65.6%)	133 (50.8%)	59 (64.1%)	2709 (64.6%)	
Fair	555 (14.5%)	89 (34.0%)	22 (23.9%)	666 (15.9%)	
Poor	34 (0.9%)	19 (7.3%)	5 (5.4%)	58 (1.4%)	
Very poor	13 (0.3%)	2 (0.8%)	0 (0.0%)	15 (0.4%)	
Missing	12 (0.3%)	2 (0.8%)	0 (0.0%)	14 (0.3%)	

*IRSAD* index of relative socio-economic advantage and disadvantage.

^a^*p*-values from Pearson’s chi-squared test.

### Self-reported and photograph-graded AMD

In response to the questionnaire, 262 (6.2%) participants reported having AMD diagnosed by a doctor and 92 (2.2%) did not know. People who reported having AMD were more likely to be older and more likely to be female than those who reported never being diagnosed (see Table 1). People who reported that they did not know if they had AMD, were more likely to have been born overseas, have a primary language other than English, have fewer years of formal education, and live in an area with less advantage/more disadvantage (see Table 1). AMD was detected on photographs of 1601 (38.2%) participants (early *n* = 867, 20.7%; intermediate *n* = 686, 16.4%; and late AMD *n* = 48, 1.1%, see Table 2).

**Table 2:** Agreement between photograph-graded and self-reported age-related macular degeneration.

Reference standard photograph-graded AMD grade	Index test “Has a doctor ever told you that you have macular degeneration?”, *n* (%)^a^			Total
	Don’t know	No	Yes	
Total	92 (2.2%)	3839 (91.6%)	262 (6.2%)	4193 (100%)
Beckman AMD stage				
None/normal ageing	55 (2.1%)	2458 (94.8%)	79 (3.0%)	2592 (100%)
Early	24 (2.8%)	805 (92.8%)	38 (4.4%)	867 (100%)
Intermediate	13 (1.9%)	570 (83.1%)	103 (15.0%)	686 (100%)
Late	0 (0.0%)	6 (12.5%)	42 (87.5%)	48 (100%)
Any-stage AMD				
No	55 (2.1%)	2458 (94.8%)	79 (3.0%)	2592 (100%)
Yes	37 (2.3%)	1381 (86.3%)	183 (11.4%)	1601 (100%)
Late AMD				
No	92 (2.2%)	3833 (92.5%)	220 (5.3%)	4145 (100%)
Neovascular only	0 (0.0%)	3 (20.0%)	12 (80.0%)	15 (100%)
Atrophic only	0 (0.0%)	3 (10.7%)	25 (89.3%)	28 (100%)
Neovascular & atrophic	0 (0.0%)	0 (0.0%)	5 (100.0%)	5 (100%)

*AMD* age-related macular degeneration.

^a^Row percentages given.

### Diagnostic accuracy

The numbers of true and false positive and negative responses under each scenario are presented in Supplementary Table 3.

The questionnaire item had poor sensitivity for any-stage AMD (11.4%, see Table 3): positive responses were recorded from only 4.4%, 15.0%, and 87.5% of those with early, intermediate and late AMD, respectively, indicating that people with late AMD were more likely to be aware of having AMD than those with earlier stages of disease. Sensitivity for any-stage AMD increased with increasing age and among those with poorer self-rated eyesight, meaning that participants with AMD in these groups were more aware of having the disease than people with AMD who were either younger or had better vision (see Table 4).

**Table 3:** Diagnostic accuracy parameters for self-reported age-related macular degeneration with 95% confidence intervals (*n* = 4193).

	Reference standard AMD grade		
	Any	Intermediate/late	Late
Prevalence of reference standard (%)	38.2 (36.7,39.7)	17.5 (16.4,18.7)	1.1 (0.9,1.5)
Sensitivity (%)	11.4 (9.9,13.1)	19.8 (16.9,22.8)	87.5 (74.8,95.3)
Specificity (%)	96.9 (96.2,97.6)	96.6 (96.0,97.2)	94.7 (94.0,95.4)
Positive predictive value (%)	69.8 (63.9,75.3)	55.3 (49.1,61.5)	16.0 (11.8,21.0)
Negative predictive value (%)	63.9 (62.4,65.4)	85.0 (83.9,86.1)	99.8 (99.7,99.9)
Positive likelihood ratio	3.75 (2.90,4.85)	5.84 (4.64,7.35)	16.49 (13.95,19.49)
Negative likelihood ratio	0.91 (0.90,0.93)	0.83 (0.80,0.86)	0.13 (0.06,0.28)
Diagnostic odds ratio	4.11 (3.13,5.38)	7.03 (5.43,9.10)	124.89 (53.78,289.70)
Area under the ROC curve	0.54 (0.53,0.55)	0.58 (0.57,0.60)	0.91 (0.86,0.96)

An inconclusive index test (response = “Don’t know”, *n* = 92) was considered negative (best-case scenario).

*AMD* colour fundus photograph-graded age-related macular degeneration, *ROC* receiver operating characteristic.

**Table 4:** Sensitivity and specificity for any-stage age-related macular degeneration within subgroups of demographic variables.

	Num with condition (Prevalence)		TP	TN	FP	FN	Sensitivity (95% CI)		*p*-value	Specificity (95% CI)		*p*-value
Total	1601/4193	(38.2%)	183	2513	79	1418	11.4	(9.9,13.1)		96.9	(96.2,97.6)	
Age at randomisation (years)												
70–74	956/2738	(34.9%)	83	1742	40	873	8.7	(7.0,10.7)	Ref	97.8	(97,98.4)	Ref
75–79	419/1000	(41.9%)	54	556	25	365	12.9	(9.8,16.5)	0.017	95.7	(93.7,97.2)	0.010
80–84	170/351	(48.4%)	31	170	11	139	18.2	(12.7,24.9)	<0.001	93.9	(89.4,96.9)	0.003
85+	56/104	(53.8%)	15	45	3	41	26.8	(15.8,40.3)	<0.001	93.8	(82.8,98.7)	0.084
Gender												
Male	687/2064	(33.3%)	71	1332	45	616	10.3	(8.2,12.9)	Ref	96.7	(95.7,97.6)	Ref
Female	914/2129	(42.9%)	112	1181	34	802	12.3	(10.2,14.6)	0.233	97.2	(96.1,98.1)	0.488
Primary language												
English	1538/4035	(38.1%)	177	2421	76	1361	11.5	(10.0,13.2)	Ref	97.0	(96.2,97.6)	Ref
Other than English	63/158	(39.9%)	6	92	3	57	9.5	(3.6,19.6)	0.628	96.8	(91.1,99.3)	0.949
Country of birth												
Australia	1222/3161	(38.7%)	143	1883	56	1079	11.7	(10.0,13.6)	Ref	97.1	(96.3,97.8)	Ref
Outside Australia	379/1032	(36.7%)	40	630	23	339	10.6	(7.7,14.1)	0.540	96.5	(94.8,97.8)	0.416
Years of education												
<9	209/546	(38.3%)	33	331	6	176	15.8	(11.1,21.5)	Ref	98.2	(96.2,99.3)	Ref
9–12	664/1698	(39.1%)	74	1004	30	590	11.1	(8.9,13.8)	0.076	97.1	(95.9,98.0)	0.268
>12	728/1949	(37.4%)	76	1178	43	652	10.4	(8.3,12.9)	0.035	96.5	(95.3,97.4)	0.112
Living situation												
At home alone	501/1182	(42.4%)	67	653	28	434	13.4	(10.5,16.7)	Ref	95.9	(94.1,97.3)	Ref
With family/friends/spouse	1096/3001	(36.5%)	115	1855	50	981	10.5	(8.7,12.5)	0.094	97.4	(96.6,98.1)	0.053
In a residential/retirement home	4/10	(40.0%)	1	5	1	3	25.0	(0.6,80.6)	0.508	83.3	(35.9,99.6)	0.166
IRSAD decile^a^												
1–5 (lower levels of advantage)	541/1363	(39.7%)	53	798	24	488	9.8	(7.4,12.6)	Ref	97.1	(95.7,98.1)	Ref
6–10 (higher levels of advantage)	1058/2823	(37.5%)	130	1710	55	928	12.3	(10.4,14.4)	0.140	96.9	(96.0,97.6)	0.787
Lives in a major city^a^												
No	499/1261	(39.6%)	53	737	25	446	10.6	(8.1,13.7)	Ref	96.7	(95.2,97.9)	Ref
Yes	1100/2925	(37.6%)	130	1771	54	970	11.8	(10.0,13.9)	0.486	97.0	(96.2,97.8)	0.665
Self-rated eyesight^a^												
Fair/good/excellent	1554/4106	(37.8%)	165	2478	74	1389	10.6	(9.1,12.3)	Ref	97.1	(96.4,97.7)	Ref
Poor/very poor	39/73	(53.4%)	16	29	5	23	41.0	(25.6,57.9)	<0.001	85.3	(68.9,95.1)	<0.001

Estimated under best-case scenario: inconclusive index test (Don’t know”) classed as negative. *P*-values refer to comparisons to the reference category calculated via interaction terms in mixed-effects logistic regression models.

*IRSAD* index of relative socio-economic advantage and disadvantage, *T* true, *F* false, *P* positive, *N* negative.

^a^Missing values for IRSAD (*n* = 7), major city (*n* = 7), and self-rated eyesight (*n* = 14).

Among those without any AMD, a high proportion (96.9%) reported no diagnosis of AMD or unsure (i.e., excellent specificity, see Table 3). This value decreased slightly with increasing age and among those with poorer self-rated eyesight, meaning the participants without AMD in these categories were slightly more likely to falsely report having AMD than younger people with better vision (see Table 4). Similar patterns were observed under the alternative reference standard diagnoses (intermediate AMD or worse Supplementary Table 4; late AMD Supplementary Table 5).

The questionnaire was only slightly better than chance at distinguishing between participants with and without any-stage AMD (area under the receiver operating characteristic curve 0.54, see Table 3). Of the 262 participants who reported having AMD, evidence of any AMD was detected among 183 (positive predictive value 69.8%), including 38 (20.8%), 103 (56.3%), and 42 (23.0%) with early, intermediate and late AMD, respectively. A negative or inconclusive response (*n* = 3931) was strongly associated with the absence of late AMD (excellent negative predictive value for late AMD). However, early and intermediate AMD were detected in 829 (21.1%) and 583 (14.8%), respectively, of those with negative/inconclusive responses, and the suboptimal negative likelihood ratio (0.91) indicates a negative/inconclusive response is associated with only a minimal decrease in the probability of having any-stage AMD compared to a positive response (i.e., the questionnaire item is not very helpful for ruling out AMD).

Similar results were found under the worst-case scenario (inconclusive index test “Don’t know” treated as a positive response) and after inconclusive cases were excluded (see Supplementary Table 6). That is, excellent specificity but poor sensitivity for any-stage AMD.

## Discussion

In this large prospective study, we found that asking participants whether a doctor had ever diagnosed them with “macular degeneration” severely underestimated the prevalence of photograph-graded AMD. Although most people with vision-threatening late-stage AMD were aware of their condition, the overwhelming majority of people with early and intermediate AMD were not. While this suggests that many healthy older Australians are not undergoing regular eye examinations, it is also possible that clinicians are not passing on information about early-stage disease when identified, or that individuals have not remembered or understood the information they have been given.

Upon diagnosis with the earlier stages of AMD, patients may be encouraged to take steps to slow disease progression such as quitting cigarettes, becoming more active, and taking dietary supplements [20]. People with intermediate AMD, in particular, should be aware of their condition so they can closely monitor their vision for signs of progression and seek timely intervention if needed to prevent severe visual impairment. Thus, it is important for those affected to be aware of having AMD, even in its early stages. We found that the proportion of people correctly identifying themselves as having AMD was lowest among younger people and those with better self-rated eyesight. These characteristics should be considered as potential sources of bias in studies that use self-reported AMD status to estimate population prevalence or to assess associations between AMD and other characteristics. Conversely, among the participants who reported having AMD, almost one third did not have any evidence of it on retinal photographs, with a greater propensity for falsely reporting AMD amongst older participants and those with poorer self-rated eyesight.

Australian residents aged 65 years and over have access to government funding for annual comprehensive ocular exams and around 97% of optometry services were fully covered by government funding during the study period [21]. While it is estimated that over 80% of older Australian adults would have had an eye examination within the previous two years, access to eyecare services can vary according to location [22]. Although we did not find strong evidence that diagnostic accuracy of self-report differed according to socio-economic factors in the current study, lower sensitivity in particular demographic groups could attenuate the estimated magnitude of effect between those demographic risk factors and AMD, such as those reported in the UK Biobank studies [23].

### Comparison to previous research

Poor diagnostic accuracy for self-report of eye conditions has been acknowledged for many years, with a sensitivity (i.e., proportion of positive reports among those with the condition) for any-stage AMD of 18% in the Beaver Dam Eye Study and 5% in the Los Angeles Latino Eye Study [7, 9]. Despite the development of therapeutic agents for nAMD and improved access to eyecare services since those studies were conducted, awareness of having AMD remains low. Like the Beaver Dam Eye Study, we found the proportion of people without AMD who correctly report their status to decrease with increasing age [7]. However, unlike previous findings, the proportion of people with AMD who were aware of the condition was greater among our older participants and those with poorer self-rated vision, i.e., among those who are more likely to have had a comprehensive eye exam.

Comparable findings were reported among adults with visual acuity worse than 6/12 within a similar population to this study [8]. However, interpretation of the estimates from that study is challenging given the incongruity between the questionnaire wording (“Age-related macular degeneration [loss of your central vision]”) and the reference standard diagnosis which included the earlier, non-vision threatening stages of AMD [8].

### Strengths and limitations

Strengths of this study include the large sample size, the prospective and standardised collection of data, photo documentation of the central retinal status, and inclusion of participants from both metropolitan and regional areas. Experienced retinal image graders provided the reference standard diagnosis based on the Beckman clinical classification for AMD [17]. We investigated the diagnostic accuracy of a questionnaire item for detecting both late-stage and any-stage AMD, unlike previous studies which have only used any-stage AMD as their reference standard [7–9].

ASPREE participants were required to see well enough to independently complete a number of written tasks, potentially deterring recruitment of people with very poor vision due to ocular pathology. However, the prevalence of AMD detected in this study is consistent with that expected in this age group, indicating that the estimates of positive and negative predictive values may be reasonably extrapolated to the wider community [24]. Although the distribution of age in this study broadly reflects that of the community, people with serious health issues were excluded from enroling in the ASPREE study and the proportion of current smokers was lower in APSREE than the wider community [3]. In addition, less than 1% of participants in the current study were Indigenous Australians. Thus, we are unable to comment on the accuracy of self-report of eye conditions among these important groups.

It is possible that additional cases of AMD would have been detected using multimodal retinal imaging, including more cases of late AMD which are more difficult to diagnose on colour photography alone [25]. However, the number of undetected AMD cases is likely to be low given the diagnostic accuracy expected from expert graders of colour fundus photographs [26].

Retinal imaging was performed prior to completing the ALSOP questionnaire for the majority of participants. Some participants with healthy eyes at the time of retinal photography may have developed signs of AMD in the interim. However, this number is expected to be small given the slowly progressing nature of the condition. Other participants may have become aware of existing macular pathology prior to completing the questionnaire due to their involvement in the ASPREE-AMD study. The ALSOP questionnaire was completed at home, allowing participants to refer to records from past ocular exams if available.

### Implications for future research

We have shown that the questionnaire item, as it is currently worded, captures some people with the earlier stages of AMD. Identification of these participants may be of interest to researchers who are investigating the underlying risk factors for AMD and its sequalae [27]. However, if self-report is being used to estimate the prevalence, causes, or burden of vision impairment due to nAMD and/or GA, then revision of the wording of the questionnaire item could be beneficial given many people with these types of AMD are aware of their diagnosis. For people who respond that a doctor has diagnosed them with AMD, the inclusion of an additional item may assist in differentiating between people who have been informed of early-stage disease and those who have experienced visual disturbance due to late AMD. Consultation with patients, clinicians, and researchers would be needed to develop and validate any new questionnaire items.

Given the suboptimal positive predictive value of self-reported AMD (i.e., the probability of actually having late AMD given positive self-report), confirmatory steps such as retinal imaging or medical record review following positive self-report may be of benefit in future epidemiological studies that lack the resources required to capture retinal images for all participants [28, 29]. However, this approach would not improve the sensitivity of the questionnaire item for detecting AMD among people who are not aware of having the condition. Linkage with medical records and administrative claims databases may be useful in determining which participants have nAMD via exploration of treatment codes [6, 30]. Diagnostic codes are not routinely collected for outpatient visits in Australia, meaning that linkage with administrative claims data would not assist in the identification of earlier-stage AMD and GA cases. Likewise, the use of patient-accessible health and pharmacy records could improve participants’ ability to accurately report medical history [31], but these approaches will only be of benefit if participants undergo regular eye examinations with accurately-recorded findings. Therefore, retinal imaging of all participants is strongly recommended for future studies that aim to rigorously investigate AMD status.

## Conclusions

AMD is asymptomatic in the early stages and many Australians are unaware of these early changes. This study highlights the need for regular eye exams among older adults to detect common eye diseases, allowing treatment to be initiated in a timely manner to avoid vision loss if appropriate. Results from studies that rely on self-report should be interpreted with caution while AMD remains underdiagnosed in the community.

## Summary

### What was known before

- Self-report of chronic ocular conditions has previously been shown to be unreliable in epidemiological studies. However, the characteristics associated with reporting errors have not been investigated in detail.
- Findings from large epidemiological studies are often used to inform policy development and allocation of resources. Therefore, it is important to quantify the potential for bias of these estimates.

### What this study adds

- There continue to be many older Australians who have undiagnosed age-related macular degeneration (AMD); those with early-stage AMD, less-advanced age, and good eyesight were less likely to be aware of having AMD.
- Self-report underestimates the prevalence of AMD, especially in those with early or intermediate stage AMD.
- Self-report has suboptimal positive predictive value for AMD (i.e., many reports of AMD are false positives), indicating that steps to confirm AMD status would be beneficial in future epidemiological studies.

### Supplementary information

Supplemental Material

## Data Availability

The datasets generated during and/or analysed during the current study are not publicly available as they are part of an ongoing longitudinal study, but may be available from the corresponding author on reasonable request pending authorisation from the ASPREE and sub-study investigators.
