# Supplementary material for: Diagnostic accuracy of self-reported age-related macular degeneration in the ASPREE Longitudinal Study of Older Persons
Source: Eye (Lond). 2023 Sep 20;38(4):698–706. doi: 10.1038/s41433-023-02754-y (PMC10920750; doi:10.1038/s41433-023-02754-y)
Supplement: Supplementary file 1 — Supplemental Material [file 41433_2023_2754_MOESM1_ESM.docx]

Diagnostic accuracy of self-reported age-related macular degeneration in the ASPREE Longitudinal Study of Older Persons

Supplementary information

**Supplementary Table 1**: Formulae and interpretation of diagnostic accuracy parameters

| **Parameter** | **Formula** | **Interpretation** |
| --- | --- | --- |
| Prevalence | $\frac{TP+FN}{TP+FN+TN+FP}$ | The proportion of participants with AMD detected on retinal imaging. |
| Sensitivity | $\frac{TP}{TP+FN}$ | The proportion of participants aware of having AMD among those who have image-diagnosed AMD |
| Specificity | $\frac{TN}{TN+FP}$ | The proportion of participants without self-report of AMD among those who have no evidence of AMD on imaging |
| Positive predictive value | $\frac{TP}{TP+FP}$ | The probability of having AMD for a participant who self-reports AMD. |
| Negative predictive value | $\frac{TN}{TN+FN}$ | The probability of not detecting AMD for a participant who does not report AMD. |
| Positive likelihood ratio | $\frac{Sensitivity}{1 - Specificity}$ | How many times more likely self-report is among participants with AMD than among those without AMD. “Rule-in” |
| Negative likelihood ratio | $\frac{1 - Sensitivity}{Specificity}$ | How much less likely the self-report of no AMD is for a person with AMD than for a person without AMD.  “Rule-out” |
| Diagnostic odds ratio | $\frac{TP/FN}{FP/TN}$ | The ratio of the odds of awareness of AMD among participants with disease relative to the odds of awareness among participants without disease |
| Area under the ROC curve | $\frac{Sensitivity+Specificity}{2}$ | Estimates discrimination power |
| FN = false negative, FP = false positive, TN = true negative, TP = true positive, ROC = receiver operating characteristic | | |

**Supplementary Table 2**: Characteristics of Australian ASPREE participants included and excluded in analyses

|  | **Retinal**  **photograph not taken** | **Ungradable image or missing self-reported AMD status** | **Included** | **Total** | **p-value*** |
| --- | --- | --- | --- | --- | --- |
|  |  |  |  |  |  |
|  | (n=11,281) | (n=1,229) | (n=4,193) | (N=16,703) |  |
| Age at randomisation (years) |  |  |  |  |  |
| Median, IQR | 74.3 (71.9-78.1) | 74.3 (71.9-78.0) | 73.3 (71.4-76.5) | 74.1 (71.8-77.7) |  |
| n (%) |  |  |  |  | <0.001 |
| 70-74 | 6,244 (55.3%) | 687 (55.9%) | 2,738 (65.3%) | 9,669 (57.9%) |  |
| 75-79 | 3,097 (27.5%) | 334 (27.2%) | 1,000 (23.8%) | 4,431 (26.5%) |  |
| 80-84 | 1,455 (12.9%) | 157 (12.8%) | 351 ( 8.4%) | 1,963 (11.8%) |  |
| 85-95 | 485 ( 4.3%) | 51 ( 4.1%) | 104 ( 2.5%) | 640 ( 3.8%) |  |
| Gender, n (%) |  |  |  |  | <0.001 |
| Male | 4,890 (43.3%) | 570 (46.4%) | 2,064 (49.2%) | 7,524 (45.0%) |  |
| Female | 6,391 (56.7%) | 659 (53.6%) | 2,129 (50.8%) | 9,179 (55.0%) |  |
| Race, n (%) |  |  |  |  | <0.001 |
| White/Caucasian | 11,133 (98.7%) | 1,193 (97.1%) | 4,145 (98.9%) | 16,471 (98.6%) |  |
| Asian | 78 ( 0.7%) | 21 ( 1.7%) | 30 ( 0.7%) | 129 ( 0.8%) |  |
| >1/other/prefer not to say | 70 ( 0.6%) | 15 ( 1.2%) | 18 ( 0.4%) | 103 ( 0.6%) |  |
| Primary language, n (%) |  |  |  |  | 0.19 |
| English | 10,892 (96.6%) | 1,175 (95.6%) | 4,035 (96.2%) | 16,102 (96.4%) |  |
| Not English | 389 ( 3.4%) | 54 ( 4.4%) | 158 ( 3.8%) | 601 ( 3.6%) |  |
| Country of birth, n (%) |  |  |  |  | 0.086 |
| Australia | 8,555 (75.8%) | 897 (73.0%) | 3,161 (75.4%) | 12,613 (75.5%) |  |
| Not Australia | 2,726 (24.2%) | 332 (27.0%) | 1,032 (24.6%) | 4,090 (24.5%) |  |
| Years of education, n (%) |  |  |  |  | <0.001 |
| < 9 | 2,144 (19.0%) | 196 (15.9%) | 546 (13.0%) | 2,886 (17.3%) |  |
| 9-12 | 5,117 (45.4%) | 518 (42.1%) | 1,698 (40.5%) | 7,333 (43.9%) |  |
| >12 | 4,019 (35.6%) | 515 (41.9%) | 1,949 (46.5%) | 6,483 (38.8%) |  |
| Missing | 1 ( 0.0%) | 0 ( 0.0%) | 0 ( 0.0%) | 1 ( 0.0%) |  |
| Living situation, n (%) |  |  |  |  | <0.001 |
| At home alone | 3,733 (33.1%) | 416 (33.8%) | 1,182 (28.2%) | 5,331 (31.9%) |  |
| With family/friends/spouse | 7,505 (66.5%) | 808 (65.7%) | 3,001 (71.6%) | 11,314 (67.7%) |  |
| In a residential/retirement home | 43 ( 0.4%) | 5 ( 0.4%) | 10 ( 0.2%) | 58 ( 0.3%) |  |
| IRSAD decile, n (%) |  |  |  |  | <0.001 |
| 1-5 (lower levels of advantage) | 5,475 (48.5%) | 370 (30.1%) | 1,363 (32.5%) | 7,208 (43.2%) |  |
| 6-10 (higher levels of advantage) | 5,765 (51.1%) | 859 (69.9%) | 2,823 (67.3%) | 9,447 (56.6%) |  |
| Missing | 41 ( 0.4%) | 0 ( 0.0%) | 7 ( 0.2%) | 48 ( 0.3%) |  |
| Lives in a major city, n (%) |  |  |  |  | <0.001 |
| No | 6,329 (56.1%) | 336 (27.3%) | 1,261 (30.1%) | 7,926 (47.5%) |  |
| Yes | 4,911 (43.5%) | 893 (72.7%) | 2,925 (69.8%) | 8,729 (52.3%) |  |
| Missing | 41 ( 0.4%) | 0 ( 0.0%) | 7 ( 0.2%) | 48 ( 0.3%) |  |
| Self-rated eyesight, n (%) |  |  |  |  | <0.001 |
| Excellent | 1,445 (12.8%) | 113 ( 9.2%) | 731 (17.4%) | 2,289 (13.7%) |  |
| Good | 6,293 (55.8%) | 426 (34.7%) | 2,709 (64.6%) | 9,428 (56.4%) |  |
| Fair | 1,793 (15.9%) | 165 (13.4%) | 666 (15.9%) | 2,624 (15.7%) |  |
| Poor | 157 ( 1.4%) | 14 ( 1.1%) | 58 ( 1.4%) | 229 ( 1.4%) |  |
| Very poor | 53 ( 0.5%) | 2 ( 0.2%) | 15 ( 0.4%) | 70 ( 0.4%) |  |
| Missing | 1,540 (13.7%) | 509 (41.4%) | 14 ( 0.3%) | 2,063 (12.4%) |  |
| Self-reported cataract history, n (%) |  |  |  |  | <0.001 |
| No | 4,855 (43.0%) | 173 (14.1%) | 2,315 (55.2%) | 7,343 (44.0%) |  |
| Yes, no history of cataract surgery | 1,749 (15.5%) | 192 (15.6%) | 724 (17.3%) | 2,665 (16.0%) |  |
| Yes, had cataract surgery | 2,802 (24.8%) | 294 (23.9%) | 1,038 (24.8%) | 4,134 (24.8%) |  |
| Don't know | 177 ( 1.6%) | 17 ( 1.4%) | 66 ( 1.6%) | 260 ( 1.6%) |  |
| Missing | 1,698 (15.1%) | 553 (45.0%) | 50 ( 1.2%) | 2,301 (13.8%) |  |
| Self-reported eye conditions, n (%) |  |  |  |  | <0.001 |
| Glaucoma | 782 ( 6.9%) | 111 ( 9.0%) | 289 ( 6.9%) | 1,182 ( 7.1%) |  |
| Age-related macular degeneration | 531 ( 4.7%) | 22 ( 1.8%) | 218 ( 5.2%) | 771 ( 4.6%) |  |
| Retinopathy/diabetic retinopathy | 41 ( 0.4%) | 10 ( 0.8%) | 29 ( 0.7%) | 80 ( 0.5%) |  |
| More than one of the above | 134 ( 1.2%) | 9 ( 0.7%) | 47 ( 1.1%) | 190 ( 1.1%) |  |
| None of the above | 7,700 (68.3%) | 314 (25.5%) | 3,610 (86.1%) | 11,624 (69.6%) |  |
| Missing | 2,093 (18.6%) | 763 (62.1%) | 0 ( 0.0%) | 2,856 (17.1%) |  |
| IRSAD = Index of Relative Socio-economic Advantage & Disadvantage * P-value from Pearson's chi-squared test | | | | | |

**Supplementary Table 3**: Frequency of true and false negative and positive reports of age-related macular degeneration

| **Scenario** | **Reference standard:** Photograph-graded AMD status | | | | |
| --- | --- | --- | --- | --- | --- |
|  | **Any-stage AMD**  None/normal ageing **vs** early, intermediate, or late AMD | | | | |
|  | **Total** | **TP** | **TN** | **FP** | **FN** |
| Best-case | 4193 | 183 | 2513 | 79 | 1418 |
| Worst-case | 4193 | 220 | 2458 | 134 | 1381 |
| Inconclusive excluded | 4101 | 183 | 2458 | 79 | 1381 |
|  | **Intermediate AMD or worse**  None/normal ageing or early AMD **vs** intermediate or late AMD | | | | |
|  | **Total** | **TP** | **TN** | **FP** | **FN** |
| Best-case | 4193 | 145 | 3342 | 117 | 589 |
| Worst-case | 4193 | 158 | 3263 | 196 | 576 |
| Inconclusive excluded | 4101 | 145 | 3263 | 117 | 576 |
|  | **Late AMD**  None/normal ageing, early or intermediate AMD **vs** late AMD | | | | |
|  | **Total** | **TP** | **TN** | **FP** | **FN** |
| Best-case | 4193 | 42 | 3925 | 220 | 6 |
| Worst-case | 4193 | 42 | 3833 | 312 | 6 |
| Inconclusive excluded | 4101 | 42 | 3833 | 220 | 6 |
| AMD = age-related macular degeneration, T = true, F = false, P = positive, N = negative Best-case scenario: inconclusive index test ("Don't know", n=92) considered negative. Worst-case scenario: inconclusive index test considered positive. | | | | | |

**Supplementary Table 4**: Diagnostic accuracy for intermediate age-related macular degeneration or worse within subgroups of demographic variables

|  | **Num with condition (Prevalence)** | | **TP** | **TN** | **FP** | **FN** | **Sensitivity (95% CI)** | | **p-value** | **Specificity (95% CI)** | | **p-value** |
| --- | --- | --- | --- | --- | --- | --- | --- | --- | --- | --- | --- | --- |
| Total | 734/4193 | (17.5%) | 145 | 3342 | 117 | 589 | 19.8 | (16.9,22.8) |  | 96.6 | (96.0,97.2) |  |
| Age at randomisation (years) |  |  |  |  |  |  |  |  |  |  |  |  |
| 70-74 | 403/2738 | (14.7%) | 64 | 2276 | 59 | 339 | 15.9 | (12.5,19.8) | Ref | 97.5 | (96.8,98.1) | Ref |
| 75-79 | 204/1000 | (20.4%) | 42 | 759 | 37 | 162 | 20.6 | (15.3,26.8) | 0.150 | 95.4 | (93.7,96.7) | 0.003 |
| 80-84 | 91/351 | (25.9%) | 25 | 243 | 17 | 66 | 27.5 | (18.6,37.8) | 0.010 | 93.5 | (89.7,96.2) | <0.001 |
| 85-95 | 36/104 | (34.6%) | 14 | 64 | 4 | 22 | 38.9 | (23.1,56.5) | <0.001 | 94.1 | (85.6,98.4) | 0.098 |
| Gender |  |  |  |  |  |  |  |  |  | 96.6 |  |  |
| Male | 313/2064 | (15.2%) | 54 | 1689 | 62 | 259 | 17.3 | (13.2,21.9) | Ref | 96.5 | (95.5,97.3) | Ref |
| Female | 421/2129 | (19.8%) | 91 | 1653 | 55 | 330 | 21.6 | (17.8,25.9) | 0.143 | 96.8 | (95.8,97.6) | 0.602 |
| Primary language |  |  |  |  |  |  |  |  |  |  |  |  |
| English | 709/4035 | (17.6%) | 141 | 3214 | 112 | 568 | 19.9 | (17,23) | Ref | 96.6 | (96.0,97.2) | Ref |
| Not English | 25/158 | (15.8%) | 4 | 128 | 5 | 21 | 16.0 | (4.5,36.1) | 0.632 | 96.2 | (91.4,98.8) | 0.806 |
| Country of birth |  |  |  |  |  |  |  |  |  |  |  |  |
| Australia | 574/3161 | (18.2%) | 114 | 2502 | 85 | 460 | 19.9 | (16.7,23.4) | Ref | 96.7 | (96.0,97.4) | Ref |
| Not Australia | 160/1032 | (15.5%) | 31 | 840 | 32 | 129 | 19.4 | (13.6,26.4) | 0.891 | 96.3 | (94.9,97.5) | 0.588 |
| Years of education |  |  |  |  |  |  |  |  |  |  |  |  |
| < 9 | 98/546 | (17.9%) | 23 | 432 | 16 | 75 | 23.5 | (15.5,33.1) | Ref | 96.4 | (94.3,98.0) | Ref |
| 9-12 | 309/1698 | (18.2%) | 58 | 1343 | 46 | 251 | 18.8 | (14.6,23.6) | 0.311 | 96.7 | (95.6,97.6) | 0.791 |
| >12 | 327/1949 | (16.8%) | 64 | 1567 | 55 | 263 | 19.6 | (15.4,24.3) | 0.402 | 96.6 | (95.6,97.4) | 0.853 |
| Living situation |  |  |  |  |  |  |  |  |  |  |  |  |
| At home alone | 233/1182 | (19.7%) | 56 | 910 | 39 | 177 | 24.0 | (18.7,30.1) | Ref | 95.9 | (94.4,97.1) | Ref |
| With family/friends/spouse | 499/3001 | (16.6%) | 88 | 2425 | 77 | 411 | 17.6 | (14.4,21.3) | 0.043 | 96.9 | (96.2,97.6) | 0.134 |
| In a residential/retirement home | 2/10 | (20.0%) | 1 | 7 | 1 | 1 | 50.0 | (1.3,98.7) | 0.419 | 87.5 | (47.4,99.7) | 0.266 |
| IRSAD decile* |  |  |  |  |  |  |  |  |  |  |  |  |
| 1-5 (lower levels of advantage) | 253/1363 | (18.6%) | 39 | 1072 | 38 | 214 | 15.4 | (11.2,20.5) | Ref | 96.6 | (95.3,97.6) | Ref |
| 6-10 (higher levels of advantage) | 480/2823 | (17.0%) | 106 | 2264 | 79 | 374 | 22.1 | (18.5,26.1) | 0.032 | 96.6 | (95.8,97.3) | 0.938 |
| Lives in a major city* |  |  |  |  |  |  |  |  |  |  |  |  |
| No | 247/1261 | (19.6%) | 42 | 978 | 36 | 205 | 17.0 | (12.5,22.3) | Ref | 96.4 | (95.1,97.5) | Ref |
| Yes | 486/2925 | (16.6%) | 103 | 2358 | 81 | 383 | 21.2 | (17.6,25.1) | 0.179 | 96.7 | (95.9,97.4) | 0.735 |
| Self-rated eyesight* |  |  |  |  |  |  |  |  |  |  |  |  |
| Fair/good/excellent | 701/4106 | (17.1%) | 128 | 3294 | 111 | 573 | 18.3 | (15.5,21.3) | Ref | 96.7 | (96.1,97.3) | Ref |
| Poor/very poor | 27/73 | (37.0%) | 16 | 41 | 5 | 11 | 59.3 | (38.8,77.6) | <0.001 | 89.1 | (76.4,96.4) | 0.008 |
| IRSAD = index of relative socio-economic advantage and disadvantage, T = true, F = false, P = positive, N = negative. Estimated under best-case scenario: inconclusive index test (“Don't know") treated as a negative response. P-values refer to comparisons to the reference category calculated via interaction terms in mixed-effects logistic regression models. * Missing values for IRSAD (n=7), major city (n=7), and self-rated eyesight (n=14). | | | | | | | | | | | | |

**Supplementary Table 5**: Diagnostic accuracy for late age-related macular degeneration within subgroups of demographic variables

|  | **Num with condition (Prevalence)** | | **TP** | **TN** | **FP** | **FN** | **Sensitivity (95% CI)** | | **p-value** | **Specificity (95% CI)** | | **p-value** | |
| --- | --- | --- | --- | --- | --- | --- | --- | --- | --- | --- | --- | --- | --- |
| Total | 48/4193 | (1.1%) | 42 | 3925 | 220 | 6 | 87.5 | (74.8,95.3) |  | 94.7 | (94.0,95.4) |  | |
| Age at randomisation (years) |  |  |  |  |  |  |  |  |  |  |  |  | |
| 70-74 | 13/2738 | (0.5%) | 12 | 2614 | 111 | 1 | 92.3 | (64.0,99.8) | Ref | 95.9 | (95.1,96.6) | Ref | |
| 75-79 | 17/1000 | (1.7%) | 14 | 918 | 65 | 3 | 82.4 | (56.6,96.2) | 0.439 | 93.4 | (91.7,94.9) | 0.001 | |
| 80-84 | 12/351 | (3.4%) | 11 | 308 | 31 | 1 | 91.7 | (61.5,99.8) | 0.953 | 90.9 | (87.3,93.7) | <0.001 | |
| 85-95 | 6/104 | (5.8%) | 5 | 85 | 13 | 1 | 83.3 | (35.9,99.6) | 0.562 | 86.7 | (78.4,92.7) | <0.001 | |
| Gender |  |  |  |  |  |  |  |  |  |  |  |  | |
| Male | 17/2064 | (0.8%) | 15 | 1946 | 101 | 2 | 88.2 | (63.6,98.5) | Ref | 95.1 | (94.0,96.0) | Ref | |
| Female | 31/2129 | (1.5%) | 27 | 1979 | 119 | 4 | 87.1 | (70.2,96.4) | 0.909 | 94.3 | (93.3,95.3) | 0.290 | |
| Primary language |  |  |  |  |  |  |  |  |  |  |  |  | |
| English | 44/4035 | (1.1%) | 38 | 3776 | 215 | 6 | 86.4 | (72.7,94.8) | Ref | 94.6 | (93.9,95.3) | Ref | |
| Not English | 4/158 | (2.5%) | 4 | 149 | 5 | 0 | 100.0 | (39.8,100.0) | 0.994 | 96.8 | (92.6,98.9) | 0.250 | |
| Country of birth |  |  |  |  |  |  |  |  |  |  |  |  | |
| Australia | 33/3161 | (1.0%) | 29 | 2958 | 170 | 4 | 87.9 | (71.8,96.6) | Ref | 94.6 | (93.7,95.3) | Ref | |
| Not Australia | 15/1032 | (1.5%) | 13 | 967 | 50 | 2 | 86.7 | (59.5,98.3) | 0.906 | 95.1 | (93.6,96.3) | 0.522 | |
| Years of education |  |  |  |  |  |  |  |  |  |  |  |  | |
| < 9 | 8/546 | (1.5%) | 7 | 506 | 32 | 1 | 87.5 | (47.4,99.7) | Ref | 94.1 | (91.7,95.9) | Ref | |
| 9-12 | 18/1698 | (1.1%) | 16 | 1592 | 88 | 2 | 88.9 | (65.3,98.6) | 0.919 | 94.8 | (93.6,95.8) | 0.527 | |
| >12 | 22/1949 | (1.1%) | 19 | 1827 | 100 | 3 | 86.4 | (65.1,97.1) | 0.935 | 94.8 | (93.7,95.8) | 0.490 | |
| Living situation |  |  |  |  |  |  |  |  |  |  |  |  | |
| At home alone | 18/1182 | (1.5%) | 16 | 1085 | 79 | 2 | 88.9 | (65.3,98.6) | Ref | 93.2 | (91.6,94.6) | Ref | |
| With family/friends/spouse | 29/3001 | (1.0%) | 25 | 2832 | 140 | 4 | 86.2 | (68.3,96.1) | 0.789 | 95.3 | (94.5,96.0) | 0.008 | |
| In a residential/retirement home | 1/10 | (10.0%) | 1 | 8 | 1 | 0 | 100.0 | (2.5,100.0) | 0.991 | 88.9 | (51.8,99.7) | 0.613 | |
| IRSAD decile* |  |  |  |  |  |  |  |  |  |  |  |  | |
| 1-5 (lower levels of advantage) | 17/1363 | (1.2%) | 13 | 1282 | 64 | 4 | 76.5 | (50.1,93.2) | Ref | 95.2 | (94.0,96.3) | Ref | |
| 6-10 (higher levels of advantage) | 31/2823 | (1.1%) | 29 | 2636 | 156 | 2 | 93.5 | (78.6,99.2) | 0.107 | 94.4 | (93.5,95.2) | 0.264 | |
| Lives in a major city* |  |  |  |  |  |  |  |  |  |  |  |  | |
| No | 17/1261 | (1.3%) | 14 | 1180 | 64 | 3 | 82.4 | (56.6,96.2) | Ref | 94.9 | (93.5,96.0) | Ref | |
| Yes | 31/2925 | (1.1%) | 28 | 2738 | 156 | 3 | 90.3 | (74.3,98.0) | 0.431 | 94.6 | (93.7,95.4) | 0.747 | |
| Self-rated eyesight* |  |  |  |  |  |  |  |  |  |  |  |  | |
| Fair/good/excellent | 39/4106 | (0.9%) | 33 | 3861 | 206 | 6 | 84.6 | (69.5,94.1) | Ref | 94.9 | (94.2,95.6) | Ref | |
| Poor/very poor | 9/73 | (12.3%) | 9 | 52 | 12 | 0 | 100.0 | (66.4,100.0) | 0.899 | 81.3 | (69.5,89.9) | <0.001 | |
| IRSAD = index of relative socio-economic advantage and disadvantage, T = true, F = false, P = positive, N = negative. Estimated under best-case scenario: inconclusive index test (“Don't know”) treated as a negative response. P-values refer to comparisons to the reference category calculated via interaction terms in mixed-effects logistic regression models. * Missing values for IRSAD (n=7), major city (n=7), and self-rated eyesight (n=14). | | | | | | | | | | | | |  |

**Supplementary Table 6**: Sensitivity analyses of diagnostic accuracy parameters for self-reported age-related macular degeneration with 95% confidence intervals

| **Parameter** | **Reference standard AMD grade** | | | |
| --- | --- | --- | --- | --- |
|  | **Any** | **Intermediate/late** | **Late** | |
| **Best-case scenario** (n = 4193) |  |  |  | |
| Prevalence of reference standard (%) | 38.2 (36.7,39.7) | 17.5 (16.4,18.7) | 1.1 (0.9,1.5) | |
| Sensitivity (%) | 11.4 (9.9,13.1) | 19.8 (16.9,22.8) | 87.5 (74.8,95.3) | |
| Specificity (%) | 96.9 (96.2,97.6) | 96.6 (96.0,97.2) | 94.7 (94.0,95.4) | |
| Positive predictive value (%) | 69.8 (63.9,75.3) | 55.3 (49.1,61.5) | 16.0 (11.8,21.0) | |
| Negative predictive value (%) | 63.9 (62.4,65.4) | 85.0 (83.9,86.1) | 99.8 (99.7,99.9) | |
| Positive likelihood ratio | 3.75 (2.90,4.85) | 5.84 (4.64,7.35) | 16.49 (13.95,19.49) | |
| Negative likelihood ratio | 0.91 (0.90,0.93) | 0.83 (0.80,0.86) | 0.13 (0.06,0.28) | |
| Diagnostic odds ratio | 4.11 (3.13,5.38) | 7.03 (5.43,9.10) | 124.89 (53.78,289.70) | |
| Area under the ROC curve | 0.54 (0.53,0.55) | 0.58 (0.57,0.60) | 0.91 (0.86,0.96) | |
| **Worst-case scenario** (n = 4193) |  |  |  | |
| Prevalence of reference standard (%) | 38.2 (36.7,39.7) | 17.5 (16.4,18.7) | 1.1 (0.9,1.5) | |
| Sensitivity (%) | 13.7 (12.1,15.5) | 21.5 (18.6,24.7) | 87.5 (74.8,95.3) | |
| Specificity (%) | 94.8 (93.9,95.7) | 94.3 (93.5,95.1) | 92.5 (91.6,93.3) | |
| Positive predictive value (%) | 62.2 (56.9,67.2) | 44.6 (39.4,50.0) | 11.9 (8.7,15.7) | |
| Negative predictive value (%) | 64.0 (62.5,65.6) | 85.0 (83.8,86.1) | 99.8 (99.7,99.9) | |
| Positive likelihood ratio | 2.66 (2.16,3.26) | 3.80 (3.13,4.61) | 11.62 (9.99,13.52) | |
| Negative likelihood ratio | 0.91 (0.89,0.93) | 0.83 (0.80,0.86) | 0.14 (0.06,0.29) | |
| Diagnostic odds ratio | 2.92 (2.34,3.66) | 4.57 (3.64,5.73) | 86 (37.15,198.92) | |
| Area under the ROC curve | 0.54 (0.53,0.55) | 0.58 (0.56,0.59) | 0.9 (0.85,0.95) | |
| **Inconclusive excluded** (n = 4101) |  |  |  | |
| Prevalence of reference standard (%) | 38.1 (36.7,39.6) | 17.6 (16.4,18.8) | 1.2 (0.9,1.5) | |
| Sensitivity (%) | 11.7 (10.1,13.4) | 20.1 (17.2,23.2) | 87.5 (74.8,95.3) | |
| Specificity (%) | 96.9 (96.1,97.5) | 96.5 (95.9,97.1) | 94.6 (93.8,95.3) | |
| Positive predictive value (%) | 69.8 (63.9,75.3) | 55.3 (49.1,61.5) | 16 (11.8,21.0) | |
| Negative predictive value (%) | 64.0 (62.5,65.6) | 85.0 (83.8,86.1) | 99.8 (99.7,99.9) | |
| Positive likelihood ratio | 3.76 (2.91,4.85) | 5.81 (4.62,7.31 | 16.12 (13.64,19.05) | |
| Negative likelihood ratio | 0.91 (0.89,0.93) | 0.83 (0.80,0.86) | 0.13 (0.06,0.28) | |
| Diagnostic odds ratio | 4.12 (3.14,5.41) | 7.02 (5.42,9.09) | 121.96 (52.52,282.92) | |
| Area under the ROC curve | 0.54 (0.53,0.55) | 0.58 (0.57,0.60) | 0.91 (0.86,0.96) | |
| AMD = colour fundus photograph-graded age-related macular degeneration; ROC = receiver operating characteristic. Best-case scenario: inconclusive index test ("Don't know", n=92) treated as a negative response. Worst-case scenario: inconclusive index test considered positive. | | | |  |
